# A Controller Design for Approaching Disabled Satellites Based on Discrete Sample Points

**DOI:** 10.3390/s22145091

**Published:** 2022-07-06

**Authors:** Peiyun Li, Yunfeng Dong, Yingjia Liew

**Affiliations:** School of Astronautics, Beihang University, Beijing 100191, China; lipeiyun@buaa.edu.cn (P.L.); liewyingjia@buaa.edu.cn (Y.L.)

**Keywords:** disabled satellites, controller design, discrete points, intelligent control

## Abstract

When approaching and removing a disabled satellite, the accuracy of the controller is imperative to the success of the mission because if the mission fails, more space debris can be produced due to satellite collision. To address this issue, a controller directly driven by discrete sample data points is proposed in this paper. First, the input vector for the controller is placed into a state space as a point. The state space also contains points constructed by the input vectors of pre-generated samples, which are created by the GPOPS planning algorithm along with control commands as sample output vectors. Then, an adjacent range is selected and the sample points within are collected. To accelerate the process, a series of data processing methods are implemented, including the dichotomy method, table look-up method, and random selection method. Finally, the control commands are computed using the iteratively reweighted least-squares algorithm with the assumption that similar inputs have similar outputs. According to the simulation results, the discrete point controller is more precise than the neural network controller.

## 1. Introduction

Capturing disabled satellites not only frees up valuable orbital resources, particularly the positions of the geostationary orbit [1], but also eliminates their potential threats to other satellites in the same orbit. In the first stage of capturing a disabled satellite, the chaser satellite follows the commands from the controller to go near the target satellite. If the control accuracy is insufficient, it can cause a collision and eventually create additional space debris. The controller should also be able to adapt to a wide range of tasks while maintaining sufficient accuracy.

Unlike traditional simple feedback control, long-range maneuver missions usually require trajectory planning, such as orbit transfer [2] and lunar landing [3]. In most cases, obtaining the optimal approach trajectory is time-consuming and costly, causing hindrance to real-time control [4]. To solve this problem, it is necessary to plan a set of trajectories in advance by using machine learning methods to create a neural network controller to achieve a real-time control [5]. This method has been applied to both orbit control and attitude control. Zhao combined neural networks with sliding mode control and applied them to spacecraft attitude control [6]. Regarding the interplanetary trajectory design, Izzo employed neural networks as surrogate planners to increase the planning speed [7]. Biggs investigated the attitude control problem using only four thrusters, in addition to the use of neural networks as real-time optimal controllers [8]. In the lunar landing problem, Sánchez-Sánchez utilized neural networks as controllers [9]. With respect to the disabled satellite capture problem, Li introduced neural networks to simultaneously control the orbit and attitude of the chaser satellite [10]. As the training of neural networks requires a large dataset and a long training time, Li suggested a meta-learning method that uses only a small dataset [11]. Existing research on neural network controllers usually utilizes the fitting ability and the fast forward propagation feature of neural networks to replace time-consuming computation and to realize real-time control. However, an output error of the neural network may lead to a decrease in control accuracy.

Compared with neural network controllers, fuzzy controllers are able to accurately generate control commands when designed according to the control inputs. The design is usually completed manually and can be easily understood. Fuzzy controllers have been widely implemented in different industries. Their applications include drying technologies [12], control problems of underactuated systems [13], multi-motor systems [14], predicting the optimization of building thermal consumption [15], humanoid robot control [16], and rotary-wing unmanned aerial vehicles [17]. The main characteristic of fuzzy control is that it makes use of a series of known anchor points, in which the relationship between the inputs and outputs is defined. The actual control decisions are made according to existing decisions that have similar control inputs, and the control decisions are usually discrete. The problem of approaching disabled satellites requires high control accuracy, for which reason the simple discrete output is inadequate.

In order to surpass the control accuracy of the current neural network control method, our method directly employed discrete points as control anchor points. The first step of the control process is to insert the control input vector into a state space as a point. After selecting the neighboring points as sample points, the iteratively reweighted least-squares algorithm is applied to obtain the control output. A series of modifications are also made to accelerate the whole process to ameliorate the time-cost trade-off.

The two key contributions of this study are:

1. Discrete sample points are directly used as reference anchor points in the control algorithm. The controller is designed according to the concept that points with similar inputs have similar outputs. This control method also fully utilizes the output values of anchor points to suppress the noise and increase the control accuracy. Another benefit of this method over neural network controllers is that it requires no training.
2. The control algorithm is specifically designed and accelerated by utilizing several methods, including the dichotomy method, table look-up method, and random selection method, which greatly reduces the computation cost.

The remaining part of this paper consists of four sections. Section 2 describes the problem formulation of the disabled satellite approach, and Section 3 details the design of the controller based on discrete sample points. Furthermore, in Section 4 tests and comparisons are made regarding the performance of the discrete point-based controller and the regular neural network controller. Lastly, the conclusions are stated in Section 5.

## 2. Problem Formulation

**Figure 1:**
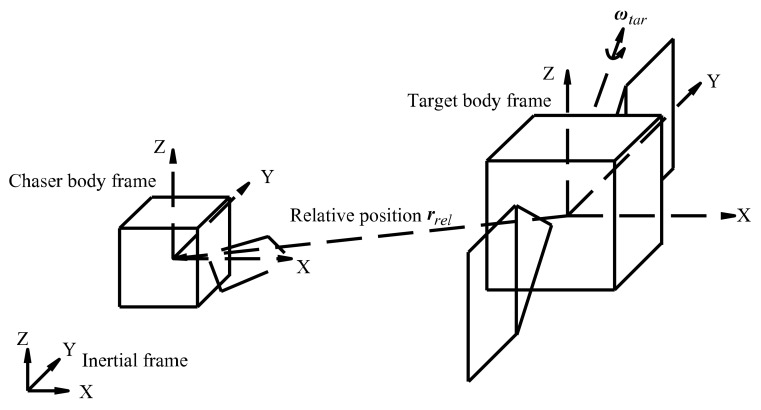
The scene of disabled satellite capture.

**Figure 2:**
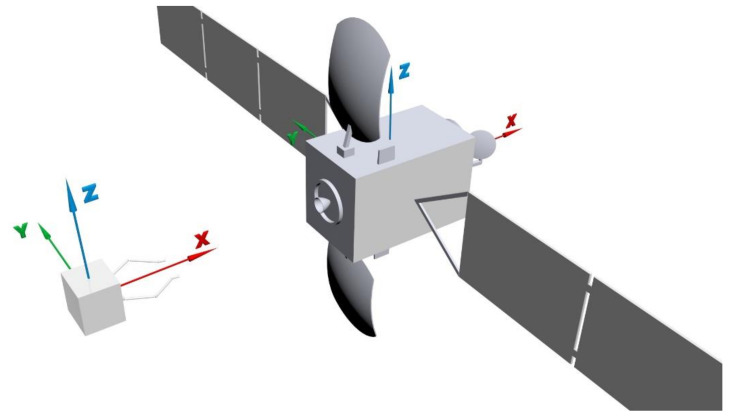
3D models of the chaser and target satellite.

As shown in Figure 1, the scene of disabled satellite capture consists of two satellites, namely the target satellite and the chaser satellite. Along its spin axis, the target satellite rotates with a certain angular velocity. The chaser satellite first departs from its initial position, then adjusts its orbital position and attitude relative to the target satellite according to the commands from the controller, remaining relatively stationary at the final time point. The origins of the chaser and target frames are located at their mass centers, which are also their geometric centers. The chaser satellite is considered a rigid body, which conforms to orbit and attitude dynamic equations [1]. The satellite 3D models are shown in Figure 2. The size of the chaser satellite is 1000 × 1000 × 1000 mm. The target satellite model in the scene is established according to the DFH-4 platform, which is the third-generation geostationary telecommunications satellite bus of China. The size of the DFH-4 platform is 2360 × 2100 × 3600 mm.

As stated in ref. [4], in the course of a chaser satellite approaching the target satellite, a state vector containing 12 elements can describe their relative motion states at every moment. Likewise, the control force and control torque of the chaser satellite at each moment can also be described by a control command vector that consists of 6 elements.

The relative state vector ***X****_rel_* ∈ **R**^1×12^ contains the relative position, velocity, Euler angle, and angular velocity. The control force and torque are combined in the control command vector ***U****_cha_* ∈ **R**^1×6^. They are denoted as

***X**_rel_* = [***r**_rel_*, ***v**_rel_*, ***A**_rel_*, ***ω**_rel_*], (1)

***U**_cha_* = [***F**_cha_*, ***T**_cha_*]. (2)

In the body frame of the chaser satellite [4], the relative position ***r****_rel_* ∈ **R**^1×3^ and relative velocity ***v****_rel_* ∈ **R**^1×3^ are defined. They can be calculated using the following equations:

***r**_rel_* = ***L**_ci_*·(***r**_tar_* − ***r**_cha_*), (3)

***v**_rel_* = ***L**_ci_*·(*****v**_tar_*** − ***v**_cha_*), (4)

where ***r****_tar_* ∈ **R**^1×3^ is the position of the target satellite in the inertial frame and ***r****_cha_* ∈ **R**^1×3^ is the position of the target satellite in the inertial frame. Transformation matrix ***L****_ci_* ∈ **R**^3×3^ represents the transformation from the inertial frame to the chaser’s body frame. It can be computed using the quaternion of the chaser satellite ***q****_cha_* [18]. Additionally, ***v****_tar_* ∈ **R**^1×3^ is the velocity of the target satellite in the inertial frame, whereas ***v****_cha_* ∈ **R**^1×3^ is the velocity of the chaser satellite in the inertial frame.

As for the relative Euler angle ***A****_rel_* ∈ **R**^1×3^, it can be obtained through the conversion from the quaternion of the chaser satellite ***q****_cha_* [1]. The definition of ***A****_rel_* is

***A**_rel_* = [***φ**_rel_*, ***θ**_rel_*, ***ψ**_rel_*]. (5)

The relative angular velocity is defined as

***ω**_rel_* = ***L**_ci_*·(***ω**_tar_* − ***ω**_cha_*), (6)

where ***ω****_tar_* ∈ **R**^1×3^ is the angular velocity of the target satellite and ***ω****_cha_* ∈ **R**^1×3^ is the angular velocity of the chaser satellite.

**Figure 3:**
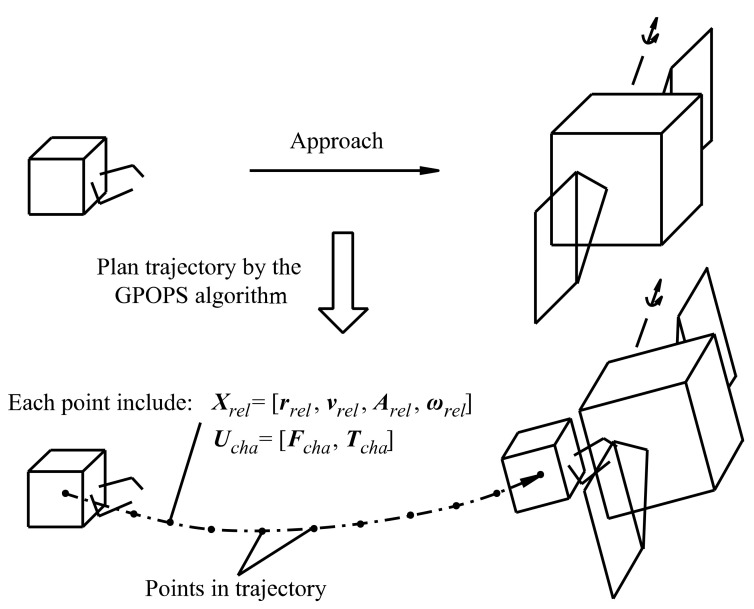
The trajectory to approach a disabled satellite.

To create a sample set for establishing the proposed discrete point controller and the neural network controller, multiple trajectories of the chaser satellite were planned by randomly selecting initial relative states. For each initial relative state, the GPOPS planning algorithm was chosen to solve the optimal control problem (see ref. [4]) and output the trajectory, as shown in Figure 3. The state vectors and their corresponding control command vectors are contained in the trajectories. 

Finally, multiple state vectors and their corresponding control command vectors were randomly selected from the trajectories to construct a sample set ***S***, which is denoted as

***S*** = [***X***, ***U***], (7)

where each row in matrix ***X*** is a state vector, which is also the sample input vector. The rows in matrix ***U*** with the same row indices are sample output vectors corresponding to the sample input vectors of matrix ***X***.

## 3. Design of Discrete Point Controller

**Figure 4:**
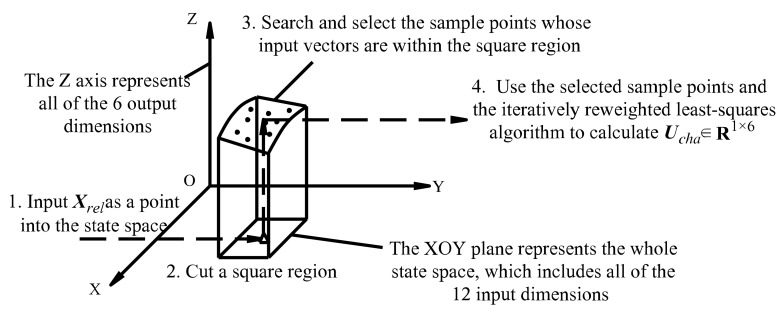
The workflow of the discrete point controller.

Differing from the common neural network controller, the controller in this paper is directly driven by discrete sample points, so it does not require the network training process. The workflow of the controller is illustrated in Figure 4. The controller takes the state vector ***X****_rel_* as an input and generates the control command vector ***U****_cha_* as an output.

The key parts of the workflow are steps 2 and 3. After the controller receives the state vector and places it into the state space, the controller cuts a square region and searches for nearby sample points that are close to the input point in all input dimensions. This approach was designed this way because we assumed that nearby sample points share similar control outputs.

**Figure 5:**
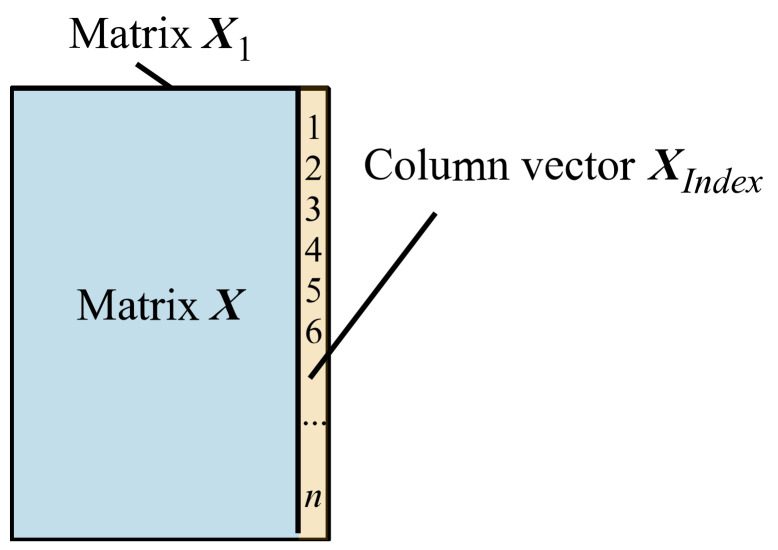
The calculation process from matrix ***X*** to matrix ***X***_1_.
- Select the data column and ID column from matrix ***X***_1_ (shown in Figure 6) and sort them by the data column in ascending order (shown in Figure 7), thereby changing the order of the ID column. As shown in Figure 8, save the sorted data columns and the sorted ID columns to matrix ***X***_2_ and matrix ***X***_3_ in the corresponding position, thereby obtaining ***X***_2_ and ***X***_3_.

One of the easiest ways to screen out the nearby points is to individually traverse all the sample points and check whether they satisfy the distance limit. However, this is much too slow to fulfill the control requirement. Therefore, in this paper, the sample format was preprocessed before applying it to the algorithm for acceleration. The preprocessing steps include:

- Specify the IDs of the sample points. After the last column of matrix ***X***, add an integer column vector ***X****_Index_* that gradually increases from 1 to *n* to obtain a combined matrix ***X***_1_, as shown in Figure 5. *n* is the row number of matrix ***X***.
  ***X***_1_ = [***X***, ***X****_Index_*]. (8)

**Figure 6:**
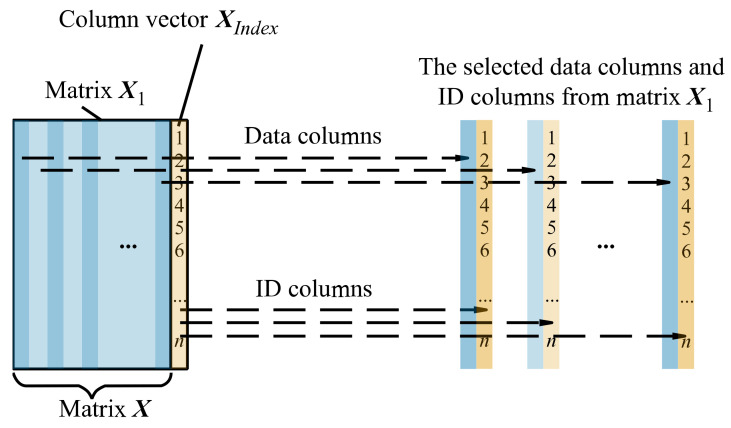
The selected data columns and ID columns from matrix ***X***_1_.

**Figure 7:**
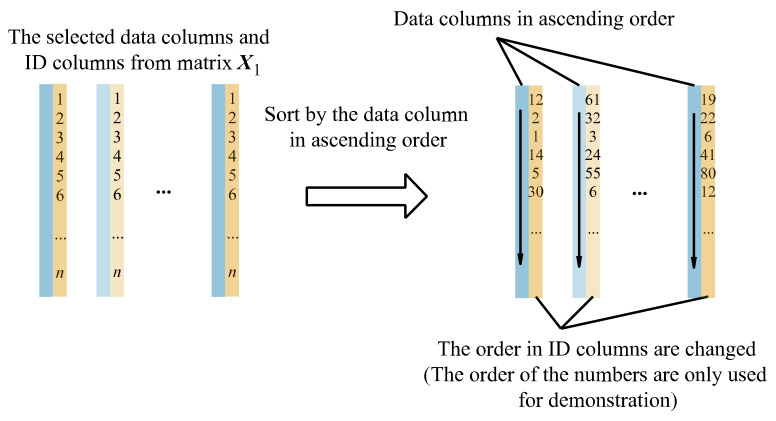
Sort the two-column matrices by the data columns in ascending order.

**Figure 8:**
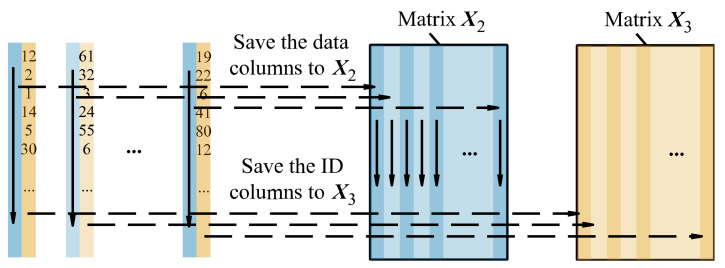
Save the sorted data columns and ID columns to matrices ***X***_2_ and ***X***_3_.
- Calculate the maximum and minimum values for each column in matrix ***X***. Store them in row vectors ***V****_max_* and ***V****_min_*.

**Figure 9:**
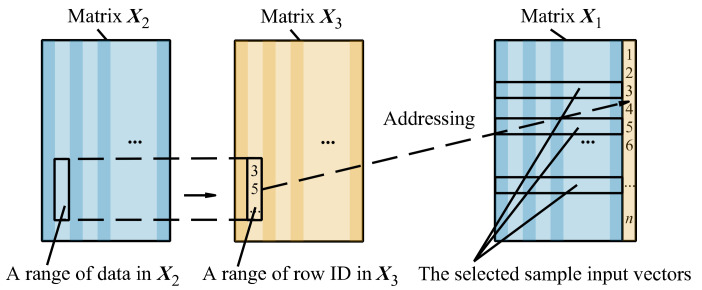
The definition and correspondence of elements in matrices ***X***_2_, ***X***_3_, and ***X***_1_.

The definition and correspondence of the elements in matrices ***X***_2_, ***X***_3_, and ***X***_1_ are shown in Figure 9.

The whole preprocessing procedure from Figure 5, Figure 6, Figure 7, Figure 8 and Figure 9 is only required to be performed once. After, based on ***X***_2_, ***X***_3_, ***V****_max_*, and ***V****_min_*, use the following steps to quickly obtain the sample points whose sample input vectors are adjacent to the actual input vector ***X****_rel_* in each dimension.

1. Specify the distance limit *r* (e.g., *r* = 5%). The limit designates a square subspace (shown in Figure 4) whose length ratio to that of the whole sample state space in each dimension is *r*. The sample points in the subspace are selected.
2. As for each of the 12 elements in the input vector, use the dichotomy method to get the range of data in ***X***_2_, as shown in Figure 10. Then, a corresponding range in ***X***_3_ and sample input vectors in ***X***_1_ can be obtained, and the sample input vector sets for each element in the input vector are generated.

**Figure 10:**
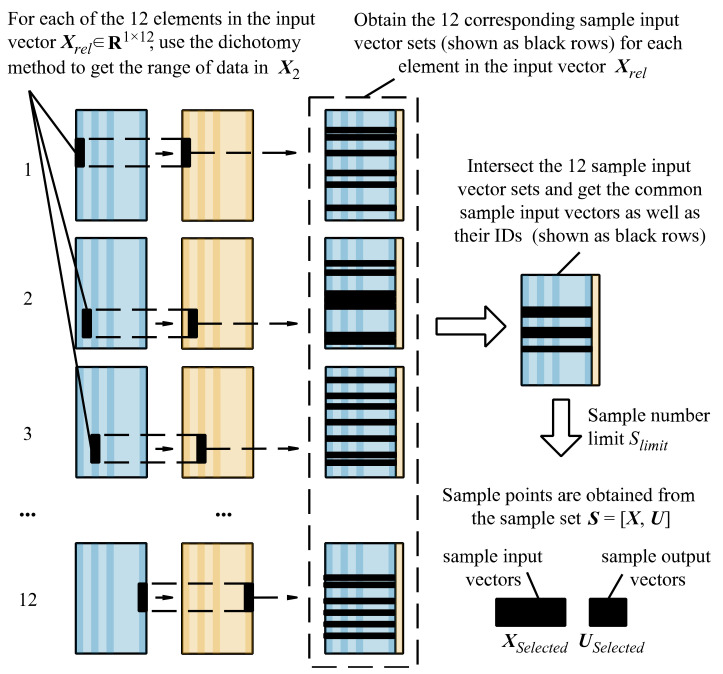
The calculation process from ***X****_rel_* to matrices ***X****_Selected_* and ***U****_Selected_*.
3. As shown in Figure 10, intersect these sample input vector sets to get the common IDs of the sample points that satisfy the distance limit *r* in all the dimensions. Then, the sample points can be obtained according to the common IDs.

Considering that excessive sample points obtained through filtration decreases the speed of the subsequent iteratively reweighted least-squares algorithm, a sample number limit *S_limit_* (e.g., *S_limit_* = 256) is introduced. When the number of sample points exceeds this value, sample points with no more than *S_limit_* are randomly selected for subsequent calculation. The filtered sample points less than *S_limit_* are saved as two matrices. The sample input vectors are saved as rows in the matrix ***X****_Selected_* and the sample output vectors are saved as rows in the matrix ***U****_Selected_*.

Lastly, denote ***U****_Selected_*(*i*) as the ith column of the matrix ***U****_Selected_* and ***U****_cha_*(*i*) as the ith element of the row vector ***U****_cha_*. The matrices ***X****_Selected_* and ***U****_Selected_* are used as the inputs of function *f* [19] to calculate the control output vector ***U****_cha_* with the following equation:

***U****_cha_*(*i*) = *f*(***X****_Selected_*,***U****_Selected_*(*i*)), *i* = 1,2,3,…,6. (9)

## 4. Result and Discussion

This section presents the testing and comparison results between the discrete point controller and the neural network controller. The target satellite is located in geostationary orbit and its initial position in the inertial frame is [42,167,000, 0, 0] m. Table 1 records the chaser’s relative position under 10 different conditions. The chaser satellite has a mass of 100 kg and its inertia matrix is diag(100, 100, 100) kgm^2^.

To generate the sample points required in the discrete point control method, the trajectories of the chaser satellite were planned by randomly selecting its initial relative states. We generated 7000 trajectories using the value ranges in Table 2 and randomly choose 100,000 sample points from these trajectories. Of the total sample points, 75% were randomly selected and used as both training neural networks and establishing discrete point controllers. The other sample points were used for testing in the form of input matrix ***I****_test_* ∈ **R**^25000×12^ and output matrix ***O****_test_* ∈ **R**^25000×6^. Each row of the two matrices represents a sample input vector and a sample output vector. After inputting each row of ***I****_test_* into the neural network controller or the discrete point controller and combining the output vectors, an output matrix ${\hat{O}}_{test}$ ∈ **R**^25000×6^ can be obtained. In Table 2, the Euler angle, angular velocity, and the relative velocity of the chaser satellite are set to zero because those variable values can be controlled and reached by the chaser satellite. It is designed to eliminate the unnecessary range of the state space and help simplify the problem of satellite approach.

**Figure 11:**
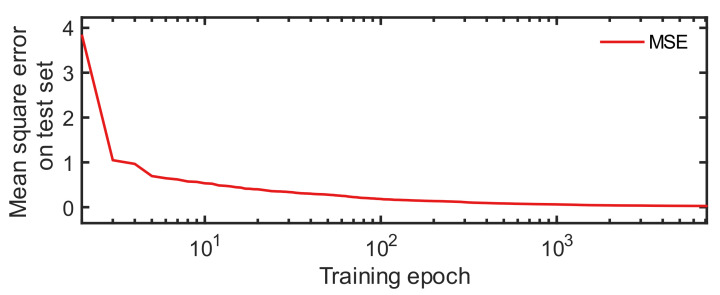
The training MSE of the optimal neural network controller.

To serve as a comparison to the proposed method, the neural network controller was also established. Since the accuracy of the neural network is affected by the activation function, the number of hidden neurons, and the training epochs, we trained a series of neural networks with different parameters and selected the one with the minimum Mean Square Error (MSE) [4] as the best neural network. The values of the parameters were: (i) the activation functions tansig and logsig, (ii) the numbers of hidden neurons chosen from [25, 31, 38, 44, 50, 57, 64, 70], and (iii) the numbers of training epochs selected from [6000, 6375, 6750, 7125, 7500]. We trained a total of 2 × 8 × 5 = 80 neural networks. After training, the chosen neural network had 25 hidden neurons and a tansig activation function. It was trained with 7125 epochs. The MSE of this neural network decreased as the number of iterations increased, as shown in Figure 11.

As for the discrete point controller, the adopted distance limit was *r* = 7%, and the sample point screening limit was *S_limit_* = 512. The mean error tested on the test sample set is also provided in Table 3, in which the error of the discrete point controller is lower. The error matrix ***O****_err_* is calculated by

(10) $$O_{err}=\left|{\hat{O}}_{test}-O_{test}\right|$$

where || denotes obtaining the absolute value for each element in a matrix. The mean error is defined as the average value of all elements in the matrix ***O****_err_*.

We compared the control performance of the discrete point controller with that of the neural network controller in the aspects of the chaser’s relative motions, which include the position, velocity, Euler angle, and angular velocity. The comparison results are presented in the tables and figures below. As can be seen in Table 4, the average position error of the discrete point controller was derived from 1.2373% = 0.08970 m/(10 − 2.75) m, where (10 − 2.75) m is the total relative distance of the chaser satellite. This suggests that the average relative position error of the discrete point controller (1.2373%) was slightly higher than that of the neural network controller (0.9306%), with a percentage difference of 0.307%. However, as can be seen from Table 5, the average relative Euler angle error of the neural network controller was [2.0912, 3.9557, 3.8625]% = [0.230, 0.435, 0.425]°/11°, where the total rotation angle of the target satellite is 11°. According to Table 5, the discrete point controller outperformed the neural network controller with an error decrease of [2.0912, 3.9557, 3.8625]% − [0.0151, 0.9654, 0.0237]% = [2.0761, 2.9903, 3.8388]%. The discrete point controller had a better overall control performance than the neural network controller.

**Figure 12:**
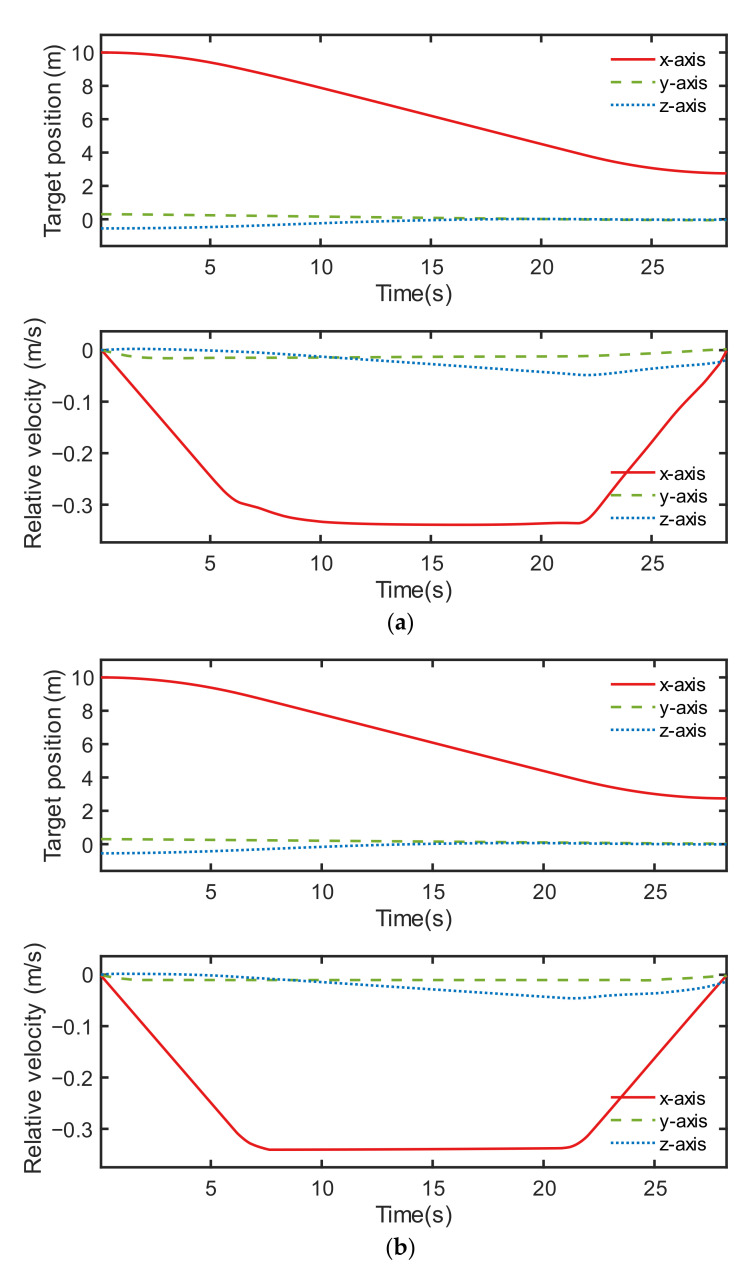
Results of orbital control, including the relative position and the relative velocity of two controllers: (**a**) neural network controller and (**b**) discrete point controller.

**Figure 13:**
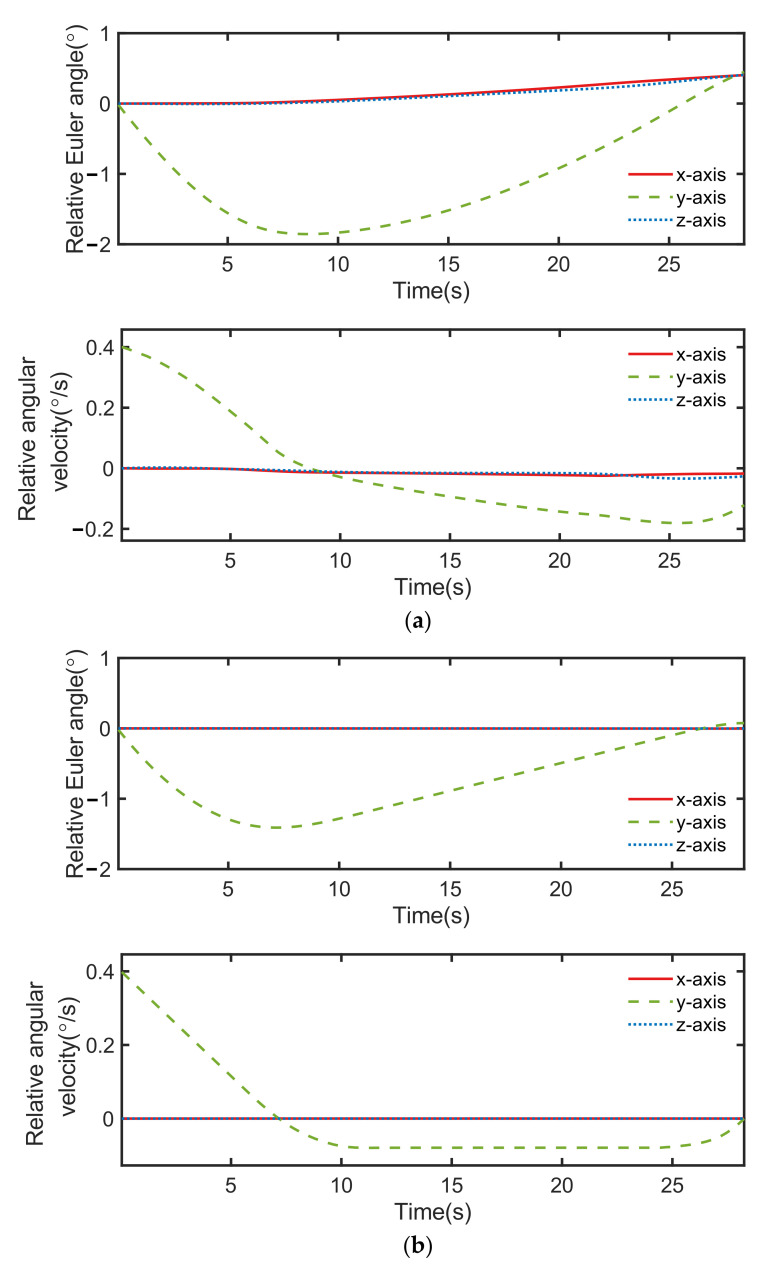
Results of attitude control, including relative Euler angle and relative angular velocity of two controllers: (**a**) neural network controller and (**b**) discrete point controller.

**Figure 14:**
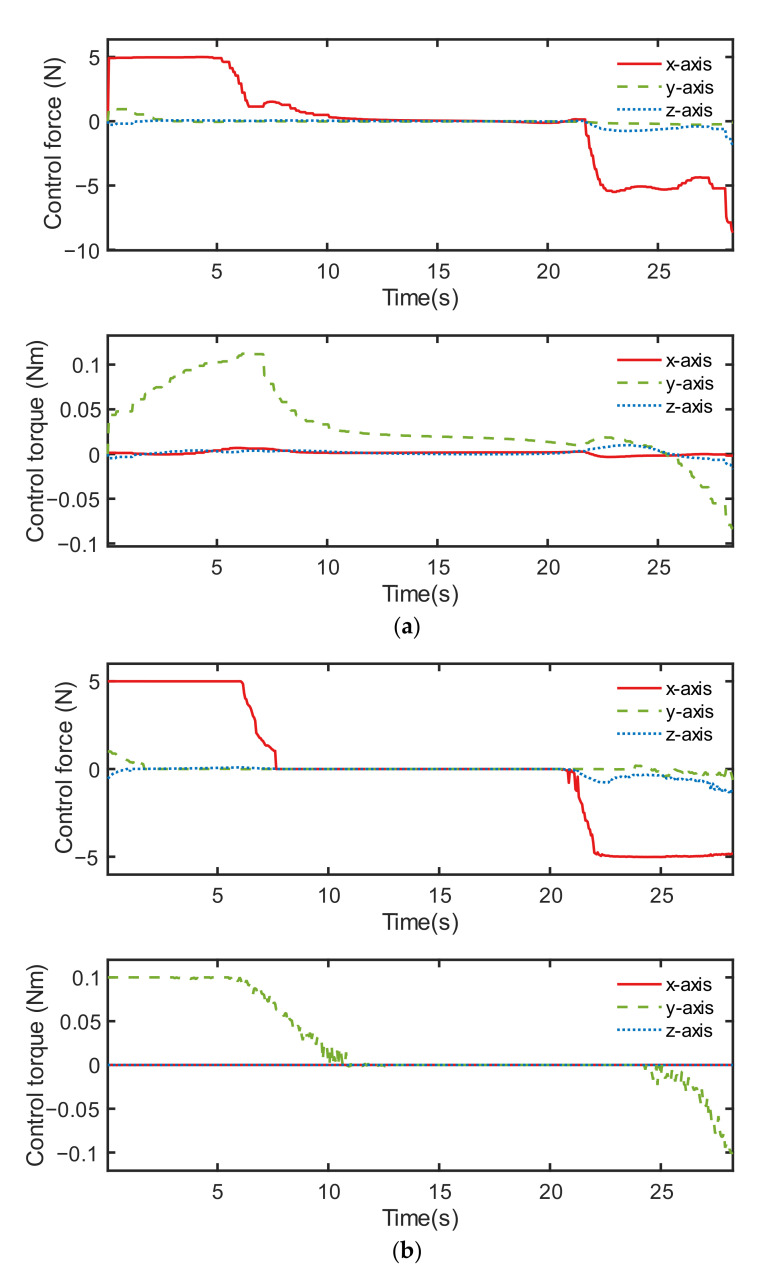
Control output, including control force and control torque of two controllers: (**a**) neural network controller and (**b**) discrete point controller.

The control performance comparison between both controllers under condition ID 6 is illustrated in Figure 12, Figure 13 and Figure 14. Although the orbital controlling performance of both controllers was relatively similar, as shown in Figure 12, Figure 13 indicates that the discrete point controller was more accurate than the neural network controller when it came to attitude control.

The control output force and output torque of the two controllers are shown in Figure 14. The discrete point controller showed better control performance, partially because its sampling is localized, and the output value is not affected by the distant sample points in the sample space. On the contrary, the neural network controller performed not so accurately, partly because its output values are influenced by a wide range of samples during the training process.

From the following tests we can see another disadvantage of the neural network controller, that is, the creation result of the neural network controller is uncertain. We applied the same training samples and training parameters: (i) the activation functions tansig and logsig, (ii) the number of hidden neurons chosen from [25, 31, 38, 44, 50, 57, 64, 70], and (iii) the number of training epochs selected from [6000, 6375, 6750, 7125, 7500]. We also trained a total of 2 × 8 × 5 = 80 neural network controllers. After training, the chosen neural network controller had 25 hidden neurons, a tansig activation function, and was trained with 7125 epochs, while the former trained neural network also had 25 hidden neurons and was trained with 7125 epochs. However, they had different parameters within their neurons.

The performance of the two neural network controllers was different, though they were trained using the same samples and were selected as the best neural networks among all parameter combinations. In Table 6 and Table 7, the two neural network controllers show differences in both orbit control performance and attitude control performance.

**Figure 15:**
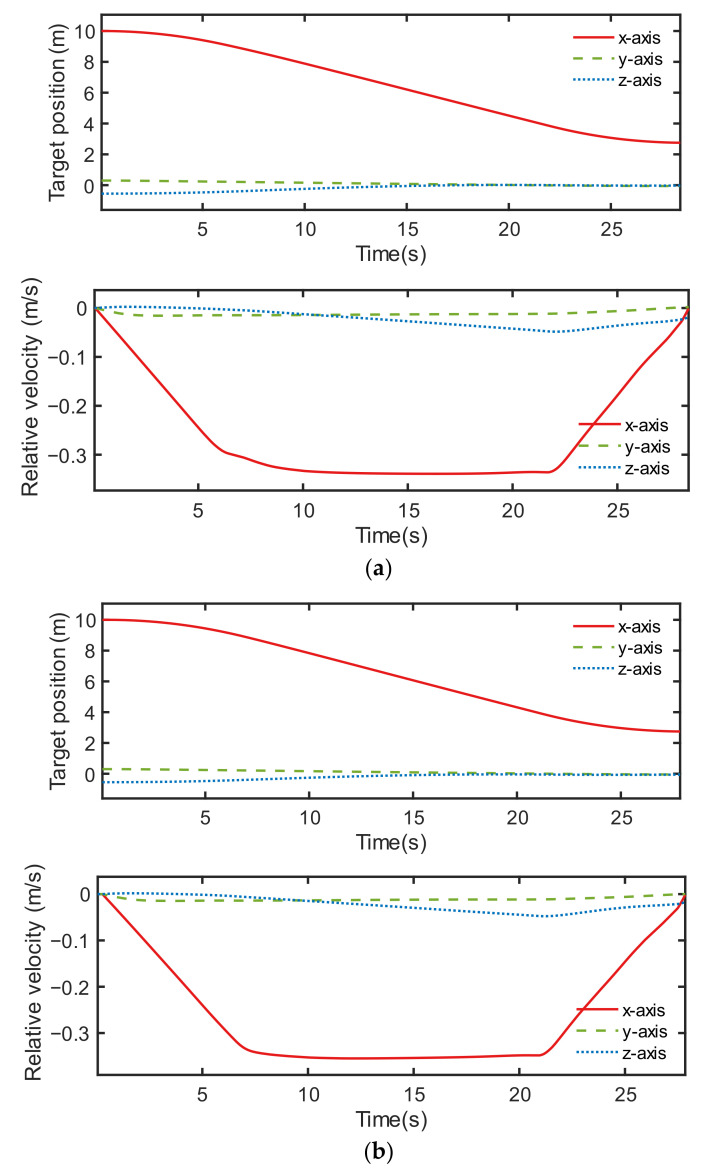
Results of orbital control, including the relative position and the relative velocity of two controllers: (**a**) neural network controller 1 and (**b**) neural network controller 2.

The test condition ID 6 was also used for the comparison of the two neural network controllers. Figure 15 illustrates that the orbit control performance did not show much difference.

**Figure 16:**
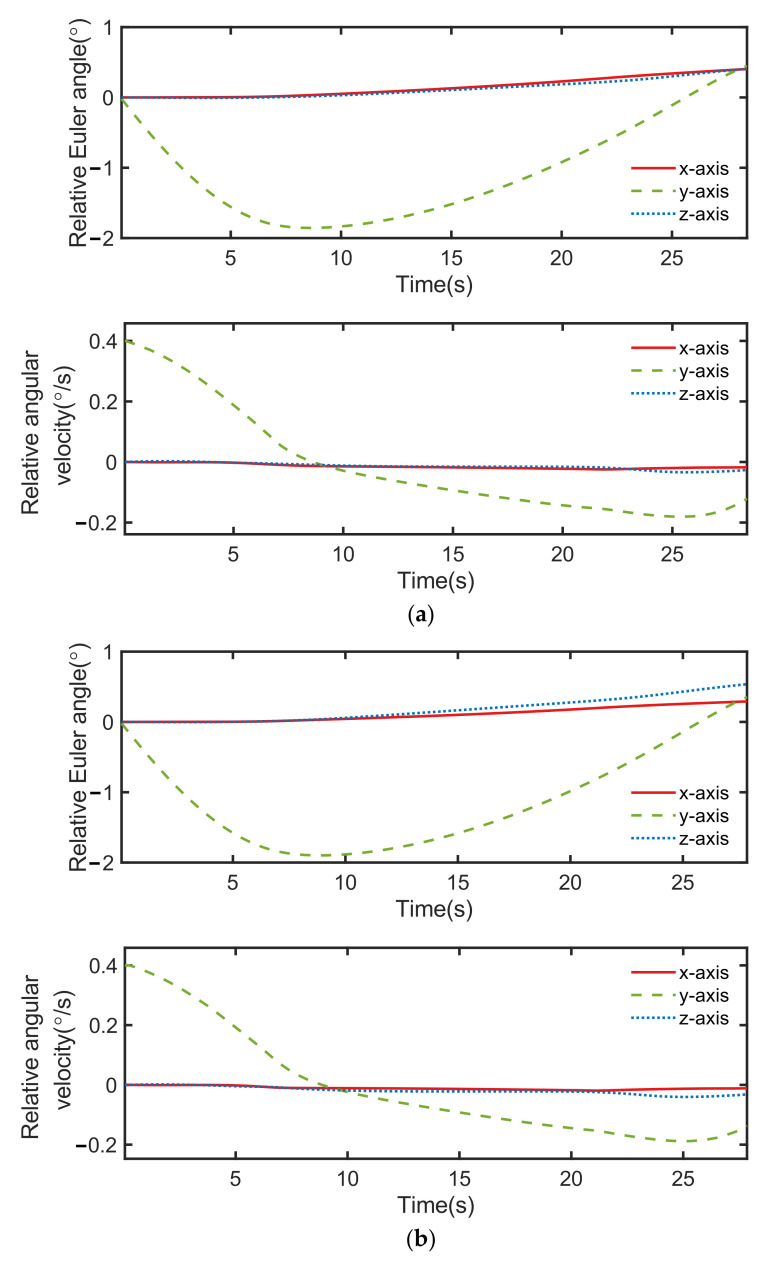
Results of attitude control, including relative Euler angle and relative angular velocity of two controllers: (**a**) neural network controller 1 and (**b**) neural network controller 2.

**Figure 17:**
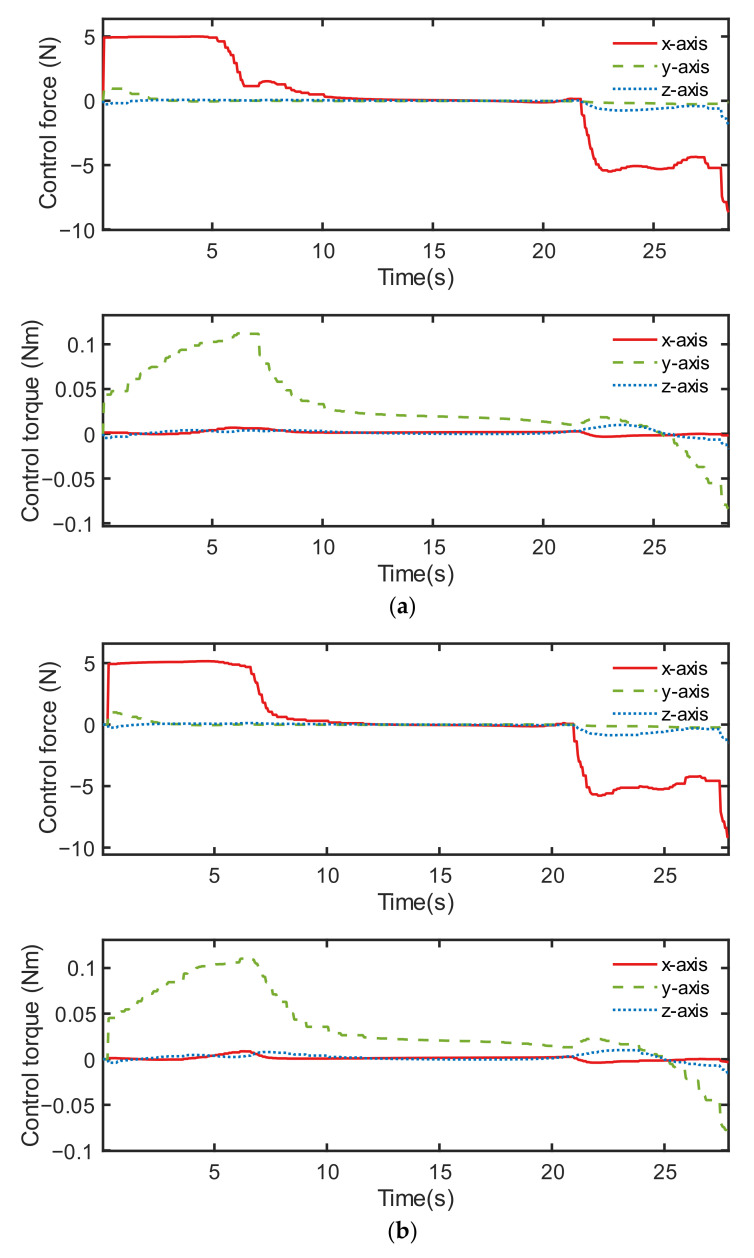
Control output, including the control force and control torque of two controllers: (**a**) neural network controller 1 and (**b**) neural network controller 2.

From Figure 16, it can be seen that the error of relative Euler angle and relative angular velocity were different in the two neural network controllers. The control commands shown in Figure 17 were also significantly changed. This is because the initialization of the neurons of the network were randomized, thus causing the difference in training results. As a result, the creation process of the neural network introduced extra uncertainty to the neural network controller.

**Figure 18:**
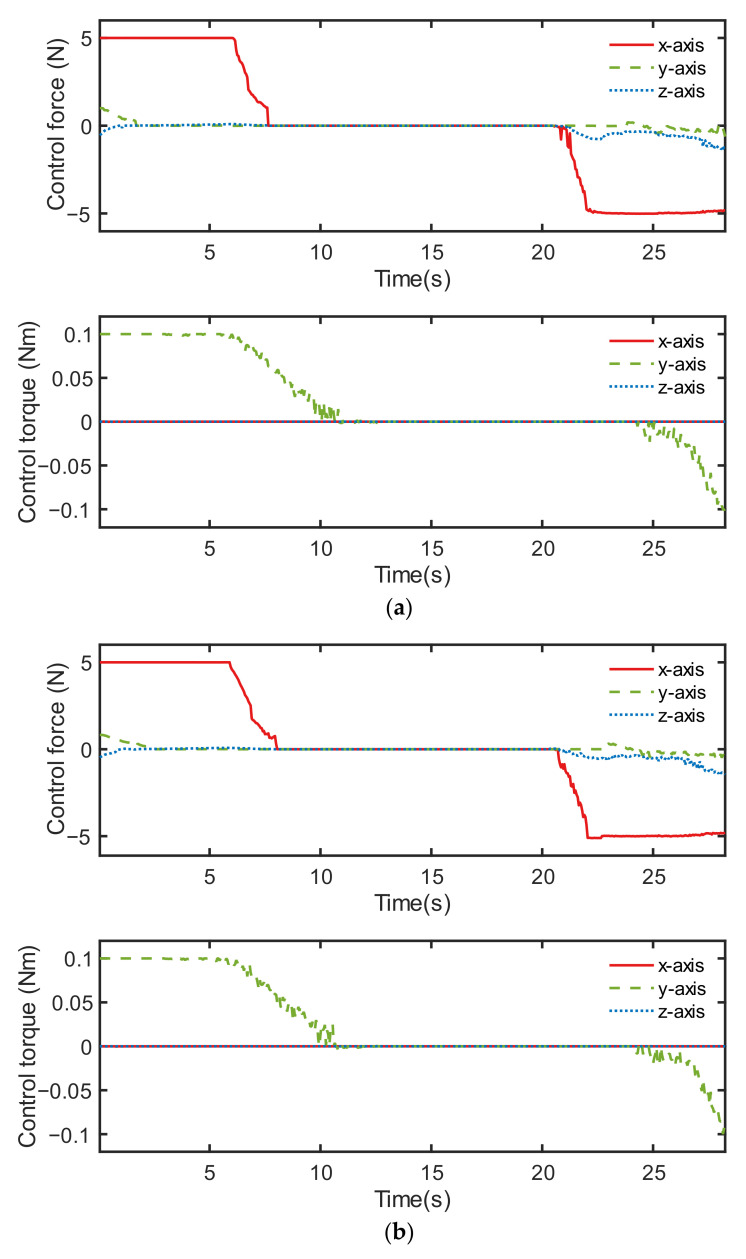
Control output, including control force and control torque of two controllers: (**a**) discrete point controller 1 and (**b**) discrete point controller 2.

We also tested the performance of the discrete point controllers with different parameters. Discrete point controller 1 was the former controller with a distance limit *r* = 7%. Discrete point controller 2 was the controller with a distance limit *r* = 9%. From Table 8 and Table 9, and Figure 18, it can be seen that the control performance and the control command curves were very similar, thus proving that the control method based on discrete points was only slightly affected by its own parameter.

Finally, the speed of our proposed method was tested using a CPU i7-8700@3.2GHz. The average time cost was 0.045 s, which is less than the general control cycle of 0.1 s.

## 5. Conclusions

In this paper, a discrete point controller design method was proposed to surpass the accuracy of neural network controllers when approaching disabled satellites. Because the proposed method directly uses the sample points, the input and output are intuitive. In the control process, the input state vectors were initially put into the input state space of the controller. Their neighboring points in each dimension were then chosen as the sample points. The iteratively reweighted least-squares algorithm was subsequently applied to these sample points to acquire the controller’s output value. Furthermore, to reduce the control computation cost, multiple methods including sample point pre-sorting, dichotomy, table look-up, and stratified random sampling, were combined and employed to accelerate the algorithm. The performance of the discrete point control method and the neural network controller were tested and compared. The simulation results suggested that the discrete point controller had more accurate working performance in various conditions, hence surpassing the neural network controller. In addition, the speed of the proposed method was tested at the end of this paper. The execution time was 0.045 s, which was generally less than the regular control period of 0.1 s.

## Figures and Tables

**Table 1:** Initial relative positions of the chaser satellite.

Condition ID	Chaser Relative Position (m)
1	[−10, 0, 0]
2	[−10, −0.55, −0.4]
3	[−10, 0.45, 0.75]
4	[−10, 0.6, 0.2]
5	[−10, −0.65, 0.45]
6	[−10, −0.3, 0.55]
7	[−10, 0.1, 0.8]
8	[−10, 0.75, 0.3]
9	[−10, −0.7, 0.1]
10	[−10, 0.3, −0.25]

**Table 2:** Range of initial relative state values for trajectory planning and simulation.

Planning Parameter	Value
Initial relative position (*x*-axis)	−10 m
Initial relative position (*y*-axis)	−2 m~2 m
Initial relative position (*z*-axis)	−2 m~2 m
Relative end position	[−2.75, 0, 0] m
Relative velocity	[0, 0, 0] m/s
Euler angle of chaser satellite	[0, 0, 0] deg
Euler angle of target satellite	[0, 0, 0] deg
Angular velocity of chaser satellite	[0, 0, 0] deg/s
Angular velocity of target satellite	[0, 0.4, 0] deg/s
Chaser control force limit	[5, 1, 1] N
Chaser control torque limit	[0.1, 0.1, 0.1] Nm

**Table 3:** Mean error of the neural network controller and the discrete point controller.

Index	Neural Network Controller	Discrete Point Controller
Mean error	4.5099%	3.0435%

**Table 4:** Orbit control performance of the two controllers under multiple working conditions. The selected time point is when the relative *x*-axis velocity reaches zero.

Orbit Control Performance	Neural Network Controller	Discrete Point Controller
Average position error of each axis	[0.0127, 0.0282, 0.0543] m	[0.0680, 0.0220, 0.0467] m
Average position error	0.06747 m	0.08970 m
Average relative position error	0.9306%	1.2373%

**Table 5:** Attitude control performance of the two controllers under multiple working conditions. The selected time point is when the relative *x*-axis velocity reaches zero.

Attitude Control Performance	Neural Network Controller	Discrete Point Controller
Average Euler angle error	[0.230, 0.435, 0.425]°	[0.0016, 0.1062, 0.0026]°
Average relative Euler angle error	[2.0912, 3.9557, 3.8625]%	[0.0151, 0.9654, 0.0237]%

**Table 6:** Orbit control performance of the two neural network controllers under multiple working conditions. The selected time point is when the relative *x*-axis velocity reaches zero.

Orbit Control Performance	Neural Network Controller 1	Neural Network Controller 2
Average position error of each axis	[0.0127, 0.0282, 0.0543] m	[0.0122, 0.0303, 0.0567] m
Average position error	0.06747 m	0.06944 m
Average relative position error	0.9306%	0.9578%

**Table 7:** Attitude control performance of the two neural network controllers under multiple working conditions. The selected time point is when the relative *x*-axis velocity reaches zero.

Attitude Control Performance	Neural Network Controller 1	Neural Network Controller 2
Average Euler angle error	[0.230, 0.435, 0.425]°	[0.229, 0.477, 0.423]°
Average relative Euler angle error	[2.0912, 3.9557, 3.8625]%	[2.0846, 4.3395, 3.8434]%

**Table 8:** Orbit control performance of discrete point controller 1 and discrete point controller 2 under multiple working conditions. The selected time point is when the relative *x*-axis velocity reaches zero.

Orbit Control Performance	Discrete Point Controller 1	Discrete Point Controller 2
Average position error of each axis	[0.0680, 0.0220, 0.0467] m	[0.0664, 0.0221, 0.0465] m
Average position error	0.08970 m	0.07286 m
Average relative position error	1.2373%	1.0050%

**Table 9:** Attitude control performance of discrete point controller 1 and discrete point controller 2 under multiple working conditions. The selected time point is when the relative *x*-axis velocity reaches zero.

Attitude Control Performance	Discrete Point Controller 1	Discrete Point Controller 2
Average Euler angle error	[0.0016, 0.1062, 0.0026]°	[0.0017, 0.9123, 0.0019]°
Average relative Euler angle error	[0.0151, 0.9654, 0.0237]%	[0.0161, 0.8294, 0.0170]%

## Data Availability

Not applicable.
